# Toll-Like Receptor Signaling in Vertebrates: Testing the Integration of Protein, Complex, and Pathway Data in the Protein Ontology Framework

**DOI:** 10.1371/journal.pone.0122978

**Published:** 2015-04-20

**Authors:** Cecilia Arighi, Veronica Shamovsky, Anna Maria Masci, Alan Ruttenberg, Barry Smith, Darren A. Natale, Cathy Wu, Peter D’Eustachio

**Affiliations:** 1 Center for Bioinformatics and Computational Biology, University of Delaware, Newark, Delaware, United States of America; 2 Department of Biochemistry & Molecular Pharmacology, NYU School of Medicine, New York, New York, United States of America; 3 Department of Immunology, Duke University, Durham, North Carolina, United States of America; 4 School of Dental Medicine, State University of New York at Buffalo, Buffalo, New York, United States of America; 5 Department of Philosophy and Center of Excellence in Bioinformatics and Life Sciences, State University of New York at Buffalo, Buffalo, New York, United States of America; 6 Protein Information Resource, Department of Biochemistry and Molecular & Cellular Biology, Georgetown University Medical Center, Washington, D. C., United States of America; SRI International, UNITED STATES

## Abstract

The Protein Ontology (PRO) provides terms for and supports annotation of species-specific protein complexes in an ontology framework that relates them both to their components and to species-independent families of complexes. Comprehensive curation of experimentally known forms and annotations thereof is expected to expose discrepancies, differences, and gaps in our knowledge. We have annotated the early events of innate immune signaling mediated by Toll-Like Receptor 3 and 4 complexes in human, mouse, and chicken. The resulting ontology and annotation data set has allowed us to identify species-specific gaps in experimental data and possible functional differences between species, and to employ inferred structural and functional relationships to suggest plausible resolutions of these discrepancies and gaps.

## Introduction

Diverse electronic databases now play central roles in storing, integrating, and analyzing information relevant to human biology. UniProt maintains definitive catalogs of the properties of human proteins and those of model organisms widely used in biomedical research [1]. Model organism databases like the Mouse Genome Database generate comprehensive catalogs of genes, functional RNAs and other genome features as well as heritable phenotypes, and curate phenotype annotations including associations of model systems with human diseases [2]. Biological pathway resources like the Reactome Knowledgebase [3] record the molecular details of processes within the human organism. These processes, decomposed into reactions, yield a network of molecular transformations that is an extended version of a classic metabolic map. Pathways identify routes connecting proteins and small molecules within the map.

Reactome and other pathway resources are rich sources of complex information curated by experts and stored in data structures developed to meet the needs of their core user communities. This richness and specialization, however, is also a limitation. The unique organization of each resource makes attempts to integrate and analyze data across resources difficult. Biomedical ontologies provide tools that can address these problems. These ontologies provide rigorous, unambiguous descriptions of biological objects and of the relationships among them using standardized and well-understood formats. Ontology structures enable the development of powerful computational tools that can reliably integrate and through both rational and statistical methods analyze the large, diverse sets of experimental data curated by independent groups of experts and stored in independent electronic databases. Within the OBO Foundry model, ontologies have been developed to describe orthogonal features of biology, but to a common standard to ensure interoperability [4]. Such ontologies link diverse structural and functional annotations into a single, coherent logical frame. Reasoning tools can identify discrepancies in represented data and suggest plausible attributes for entities that have not been experimentally studied.

GO, the Gene Ontology [5, 6], provides structured controlled vocabularies of biological terms that describe the molecular functions of gene products, their roles in biological processes, and their organization into cellular components. PRO, the Protein Ontology [7–10], captures the gene products themselves, including evolutionary families of proteins and, within each family, canonical and modified forms of proteins (“proteoforms”), the complexes they form, and their relationships. These PRO annotations link canonical species-independent forms of these entities to species-specific forms and variants.

In this work, we propose that PRO can aid the integration of disparate data and enable biologically sound inferences. As a proof-of-concept, we analyzed innate immune signaling data from different organisms (human, chicken and mouse) and sources (Reactome and Center for Computational Immunology). We studied whether Reactome’s annotations for human and chicken proteins and complexes involved in innate immune signaling [11] can be imported into formal annotations of proteins and complexes in PRO in a way that supports inferences of complex formation, subcellular localization, and roles in biological processes for corresponding mouse proteins catalogued by the Center for Computational Immunology [12].

The innate immune systems of humans and mice have both been extensively characterized so this exercise has allowed us to test the reliability of annotations in one species for predicting complex formation, subcellular location, and function in the other, and to identify true differences in the signaling processes between the two species. Where experimental data exist only for one species, we have asked whether the PRO evolutionary family framework supports plausible inferences to fill gaps.

### The innate immune signaling system

The innate immune system is an evolutionarily ancient signaling mechanism that provides an initial defense against invading microorganisms. Pattern recognition receptors (PRRs) expressed either on the cell surface or in the cytoplasm recognize microbe-associated molecular patterns (MAMPs) [13–15]. MAMP binding to a PRR triggers a signaling cascade that can result in the production of cytokines and other molecules that mediate inflammation. Several well-conserved PRR families have been identified [16]. Of these, the TLR family is the best characterized in terms of known ligands and downstream signaling pathways [17–20]. The first member of the Toll gene family was identified in *Drosophila* and shown to play a role in embryonic dorsal-ventral patterning [21]. The Drosophila Toll gene family was later shown to be critical for anti-fungal and antibacterial responses [22, 23]. Homologs of the *Drosophila* Toll protein have been identified in many other species.

### TLR Protein Family

The TLR protein family (PRO PR_000001096) contains six subfamilies with distinct ligand specificities and signaling properties [12, 24, 25]. Despite a wide range of ligands, TLRs share common structural features: a large extracellular domain (ECD), a transmembrane domain and a cytoplasmic Toll/Interleukin 1 receptor (TIR) domain. The ECD in turn consists of a varying number of leucine-rich repeats (LRR) and is responsible for MAMP recognition. The TIR domain (Pfam PF01582) interacts with downstream proteins when the ECD is activated by MAMP binding. Phylogenetic analysis of ECDs suggests that these sequences have evolved relatively rapidly in a process driven by the positive selection imposed by changing microorganisms, while TIR domains have evolved more slowly under purifying selection. TIR domains appear to have co-evolved with the intracellular adaptor molecules with which they interact [12].

**Fig 1:**
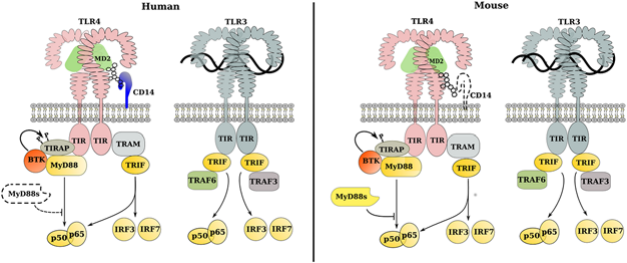
Signaling cascades initiated by ligand binding by TLR3 and TLR4 in human (left) and mouse (right). Proteins are shown as colored shapes, LPS as a cluster of open circles, and dsRNA as intertwined black lines. Entities and interactions for which there is not consistent evidence of conservation between species are shown as dotted outlines in the species for which there is no evidence for their function.

For this study, we have focused on initial steps of the signaling cascades initiated by interactions of the well-studied TLR3 and TLR4 receptors with their ligands (Fig 1). These receptors share common steps in the signaling cascade but are distinct in complex composition and the initial steps of signaling. TLR3 is associated with endosomal membranes and is implicated in the recognition of intracellular viral dsRNA. TLR4 is associated with the plasma membrane and is predominantly activated by extracellular lipopolysaccharide (LPS) derived from bacteria. Both TLR3 and TLR4 utilize TIR-domain-containing adapter-inducing interferon-β (TRIF) to signal from the endosomal compartment. TRIF-mediated signaling is essential for IFN regulatory factor (IRF)-dependent production of type I IFN. While TLR3 signals exclusively through adaptor TRIF, TLR4 can also utilize myeloid differentiation primary response 88 protein (MyD88) from its plasma membrane location. The MyD88-dependent pathway is shared by all TLR receptors except TLR3, leading to production of proinflammatory cytokines.

## Methods

PRO captures continuant properties of proteins and protein complexes such as the covalent modifications that differentiate the modified forms of a protein from one another and the identities and numbers of copies of the components of a protein complex [7, 8]. To describe the roles of proteins and complexes in the biological transformations that make up a pathway, however, it is also necessary to capture their occurent properties: molecular functions which these proteins exercise, the biological processes in which they participate and the subcellular locations which they may occupy.

Previous work within the Reactome project [11] and under the auspices of the Center for Computational Immunology [12, 26] has yielded catalogs of human, mouse, and chicken proteins and complexes involved in TLR signaling. Reactome annotations have also associated functions and subcellular locations with these proteins. PRO terms have been generated for entries in these catalogs and they have been cross-referenced to entries in Reactome, to the canonical forms of proteins in UniProt, and to entries for small molecules in CHEBI [27]. Annotated reactions and associated input and output physical entities are compiled in the supporting information associated with this paper (S1 Table); PRO, Reactome, UniProt and CHEBI terms for physical entities are shown in Tables 1 and 2.

**Table 1:** TLR3 and TLR4 complexes.

parent PRO ID	name	human PRO ID	human Reactome ID	mouse PRO ID
PR:000037302	viral dsRNA:TLR3 complex	PR:000037303	REACT_7159	PR:000037304
PR:000037306	ticam1:viral dsRNA:TLR3 complex	PR:000037307	REACT_7381	PR:000037308
PR:000037309	traf3:ticam1:activated TLR3 complex	PR:000037310	REACT_124037	PR:000037311
PR:000037343	traf6:ticam1:activated TLR3 complex	PR:000037344	REACT_25948	PR:000037471
PR:000036003	MD2:TLR4 complex	PR:000036004	REACT_7105	PR:000036005
PR:000036076	MD2:LPS:TLR4 complex	none	none	PR:000036077
PR:000025497	lipopolysaccharide receptor complex 3	PR:000025773	REACT_124771	PR:000037476
PR:000025498	lipopolysaccharide receptor complex 4	PR:000037479	REACT_124771	none
PR:000027202	ticam2:activated TLR4 complex	PR:000028678	REACT_7083	PR:000027204
PR:000027205	ticam1:ticam2:activated TLR4 complex	PR:000027208	REACT_7861	PR:000027207
PR:000028681	traf3:ticam1:ticam2:activated TLR4 complex	PR:000036022	REACT_124037	PR:000028683
PR:000028679	traf6:ticam1:ticam2:activated TLR4 complex	PR:000028680	REACT_25948	PR:000035710
PR:000036078	TIRAP:PIP2:activated TLR4 complex	PR:000036135	REACT_152404	PR:000027196
PR:000037472	TIRAP:PIP2:BTK:activated TLR4 complex	PR:000037447	REACT_124673	PR:000037477 ^+^
PR:000037472	pTIRAP:PIP2:BTK:activated TLR4 complex	PR:000037448	REACT_125282	PR:000027217
PR:000037488	MyD88:TIRAP:PIP2:BTK:activated TLR4 complex	PR:000037446	REACT_7694	PR:000037481 ^+^
PR:000025784	MyD88:Mal:activated TLR4 receptor	none	none	PR:000027174

^+^ All PRO annotations are based on experimental evidence (Evidence code ontology ECO:0000269) except ones marked with asterisks, which are based on reconstruction of a biological system (ECO:0000088)

For each complex involved in the initial steps of TLR3 or TLR4 signaling (Table 1), the PRO identifier of its species-agnostic form (parent PRO ID) is listed, together with its PRO name and the PRO identifiers of its human and mouse forms and the Reactome identifier of its human form. A version of this table with hyperlinks to the databases embedded in each identifier is available in S2 Table.

**Table 2:** TLR3 and TLR4 complex components.

parent PRO ID	Name	UniProtKB (human) or ChEBI	PRO ID human	UniProtKB (mouse) or ChEBI	PRO ID mouse
none	dsRNA	CHEBI:67208	none	CHEBI:67208	None
none	lipopolysaccharide	CHEBI:16412	none	CHEBI:16412	None
none	1-phosphatidyl-1D-myo-inositol 4,5-bisphosphate	CHEBI:18348	none	CHEBI:18348	None
PR:000025492	Toll-like receptor 4 isoform 1, signal peptide removed glycosylated 1	O00206-1	PR:000025787	Q9QUK6-1	PR:000027172
PR:000018357	Toll-like receptor 3, signal peptide removed form	O15455	PR:000037305	Q99MB1	PR:Q99MB1
PR:000003299	lymphocyte antigen 96 isoform 1, signal peptide removed, glycosylated 1	Q9Y6Y9-1	PR:000025786	Q9JHF9-1	PR:000027171
PR:000001749	TIR domain-containing adapter molecule 1 (TICAM1)	Q8IUC6	PR:Q8IUC6	Q80UF7	PR:Q80UF7
PR:000002289	TNF receptor-associated factor 3 (TRAF3)	Q13114	PR:Q13114	Q60803	PR:Q60803
PR:000002292	TNF receptor-associated factor 6	Q9Y4K3	PR:Q9Y4K3	P70196	PR:P70196
PR:000001750	TIR domain-containing adapter molecule 2	Q86XR7	PR:Q86XR7	Q8BJQ4	PR:Q8BJQ4
PR:000001740	myeloid differentiation primary response protein MyD88	Q99836	PR:Q99836	P22366-1	PR:000025766
PR:000024846	myeloid differentiation primary response protein MyD88 isoform 2	none	none	P22366-2	PR:000025767
PR:000001751	Toll/interleukin-1 receptor domain-containing adapter protein	P58753	PR:P58753	Q99JY1	PR:Q99JY1
PR:000027213	Toll/interleukin-1 receptor domain-containing adapter protein phosphorylated form	P58753	PR:000027214	none	None

For each of the three nonprotein molecules involved in forming TLR3 and TLR4 complexes, its name and identifier in the ChEBI reference database is given. For each of the proteins involved in these complexes, PRO name and the UniProt and PRO identifiers for its mouse and human forms are given. A version of this table with hyperlinks to the databases embedded in each identifier is available as S3 Table.

Both Reactome and the Center for Computational Immunology provided tab-delimited files of complexes, components and functional annotations which were used as the starting point to create PRO terms for complexes. A PRO curator reviewed the evidence for the complexes and their components and also aligned the equivalent complexes between human, mouse and chicken. The PRO curator also i) mapped the complexes to the most appropriate GO protein complex term as a parent, or created PRO complex terms to link the complexes when needed; and ii) added PRO terms for all the complex components when these were not in the ontology. The details of the curation protocol used in this work are available online [28]. Any discrepancy was reported back to the groups, re-evaluated, and resolved. The final content in PRO for the TLR set has been agreed between the different parties.

To annotate GO molecular function, biological process, and cellular location properties of these proteins and complexes in the PRO framework, we have also used relations from the OBO Foundry Relation Ontology (RO) [29, 30] in the PRO framework [7–9].

### Function

To annotate functions of instances of proteins and complexes we associate PRO terms for these entities and GO terms for molecular functions with the RO relation *has_function*. For example:

CD14 (PR:000035703) has_function LPS binding (GO:0001530);

Generalizing,

Entity1_PRO_ has_function GO:#######_GO_

where Entity1 is a protein or complex annotated in PRO and GO:####### is a molecular function term defined in GO.

**Fig 2:**
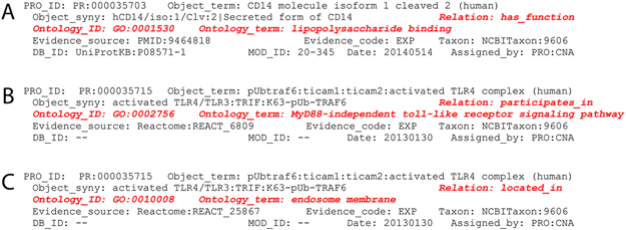
PRO stanzas illustrating the annotation of occurent properties of proteins and complexes. **A**, function; **B**, biological process; **C**, location. Stanzas are in PAF format as described previously [7]; phrases to capture function, process, and location annotations are highlighted in red.

This assertion is incorporated into the PRO PAF entry for a modified CD14 isoform as shown in Fig 2A.

Instances of complexes are annotated in the same way as individual proteins. For example:

IRF7-P:IRF7-P complex (human) (PR:000027086) has_function sequence-specific DNA binding transcription factor activity (GO:0003700).

Here and below, complexes are named by listing their constituent proteins separated by colons [31].

In addition, the PRO framework enables representation of molecular functions of components of a complex having distinct roles within the complex by creation of a term for the subtype of protein that is part of such a complex,

Complex1_PRO_ has_component Protein1_PRO_ AND Protein1_PRO_ has_function GO:#######_GO_

### Process

To annotate the involvement of instances of TLR proteins and complexes in signaling processes, PRO terms for entities are associated with GO biological process terms with the RO relation participates_in. For example (Fig 2B),

traf6:ticam1:activated TLR3 complex (human) (PR:000037344) participates_in MyD88-independent toll-like receptor signaling pathway (GO:0002756)

### Location

Cellular localization is annotated by relating the PRO term for a physical entity to a GO cellular component term. For example (Fig 2C),

IRF7 unphosphorylated 1 (PR:000037791) located_in cytoplasm (GO:0005737). Similarly, IRF7-P:IRF7-P complex (human) (PR:000027086) located_in nucleoplasm (GO:0005654).

While this is an ontological assertion about a cellular entity rather than about a protein type, inclusion of this assertion allows the ontology to be queried to identify the cellular compartment or compartments in which a process occurs.

We then use the RO relations has_component and has_part, already implemented in PRO, to form triples that relate macromolecular complexes to their component proteins and to relate proteins with their domains, respectively. For example, ticam1:viral dsRNA:TLR3 complex (mouse) (PR:000037308) has_component PR:Q80UF7 {cardinality = "2"}! TIR domain-containing adapter molecule 1 (mouse) [complex:protein], and Toll-like receptor (PR:000001096) has_part TIR domain (PF01582) [protein:domain].

The PRO terms and annotations related to this paper have been collected in a separate set of TLR-specific files, available via FTP [32]. All terms and annotations are also part of PRO release 43 and later.

The organization and content of the PRO annotation file (PAF) have been described previously [7]. Briefly, the PAF shows the annotation of PRO entities using GO or other ontologies, and adopts the format of the GO annotation file with some modifications. The PAF annotations connect PRO terms to terms from these ontologies and include the corresponding relation. Additional columns account for sequence coordinate specifications, such as the range of the sequence (for cleaved forms) or sites of covalently modified residue(s). In addition to the qualifiers used by GO (like NOT), the PAF introduces the qualifiers increased and decreased, along with a column to indicate what the object of comparison is. PAF documentation is available [33].

## Results and Discussion

Here we describe strategies to integrate PRO annotations for complexes [8] with functional annotations derived from pathway databases like Reactome and other resources, focusing on the initial steps of the TLR3 and TLR4 signaling pathways in human, mouse, and chicken.

TLR3 and TLR4 together represent key signaling strategies used by Toll receptors to initiate reactions of innate immunity. TLR4 is unique in that upon activation it recruits adaptor molecules for both MyD88-dependent and MyD88-independent signaling. TLR3 specifically uses the TRIF-signaling pathway but without the use of TRAM (Fig 1) [34]. All other TLRs activate MyD88-dependent signaling only.

Experimental studies of chicken, mouse and human systems have established that in all three species the TLR3-mediated signaling pathway is triggered by recognition of viral dsRNA and the TLR4-mediated signaling pathway is triggered by recognition of bacteria-derived LPS [35, 36]. The initial pathway steps in which a TLR receptor binds its ligand and then interacts via its cytosolic domain with its first downstream target have been annotated (S1 Table). Many of the annotations of individual proteins and complexes (Tables 1 and 2) are based on experimental observations; the rest are inferences based on relationships between the experimentally characterized proteins and their uncharacterized but structurally similar orthologues. In the course of this work, 603 new PRO terms were created, 20 for families, 64 for genes, 110 for organism-specific forms of genes, 69 for covalent modifications, 42 for organism-specific covalent modifications, 48 for GO complexes, 109 for PRO complexes, 119 for organism-specific PRO complexes, and 50 with Reactome cross-references.

**Fig 3:**
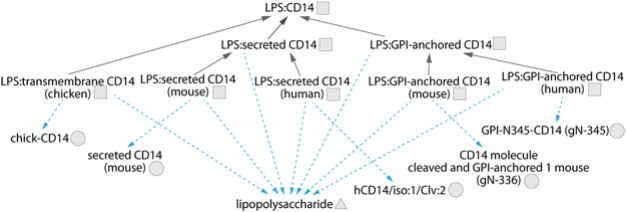
Cytoscape views of the LPS:CD14 complex repertoire. Nodes are physical entities. Circles denote proteins, triangles denote other molecules, and squares denote complexes. Dashed edges denote has_component relationships between entities; solid ones denote is_a relationships between specific and generic forms of entities.

In vertebrates the sensing of LPS involves transfer of LPS monomers to CD14 mediated by the LPS-binding protein (LBP). CD14 in turn commonly delivers the LPS to a complex of myeloid differentiation protein-2 (MD2) and TLR4 which transduces the signal through the recruitment of adaptor proteins to the TIR domain of TLR4 [34, 37]. There are three versions of the LPS:CD14 complex, namely GPI-anchored CD14:LPS, soluble CD14:LPS and transmembrane CD14:LPS (Fig 3). Each of these complexes features a distinct form of CD14. Mammals express two of these forms, soluble and GPI-anchored, whereas in birds only a complex with the transmembrane version of CD14 has been identified to date [38].

Downstream signaling complexes such as the MD2:TLR4 complex show another interesting difference between taxa. Whereas the mammalian version participates in both the MYD88-dependent and independent signaling pathways, the chicken version may only be able to participate in the MYD88-dependent pathway [34, 39]. This functional difference is captured in PRO annotations as shown in Table 3, illustrating the use of PRO annotations as a tool for making discoveries.

**Table 3:** Annotation of species-specific functions of MD2:TLR4 complexes.

MD2:TLR4 complex	Annotation	Evidence
Mouse PR:000036005	participates_in Toll-like receptor 4 signaling pathway (GO:0034142)	[52]
Human PR:000036004	participates_in Toll-like receptor 4 signaling pathway (GO:0034142)	REACT_6894
Chicken PR:000037473	NOT participates_in MyD88-independent toll-like receptor signaling pathway (GO:0002756)	[34,39] REACT_25089

A version of this table with hyperlinks to the databases embedded in each identifier is available as S4 Table.

Comparison of the PRO annotations for mouse and human CD14 complexes identified a potentially significant gap in our understanding of CD14 function (Table 1; Fig 1). In well-studied mouse and human systems, CD14 binds LPS and brings it in close proximity to the TLR4:MD2 complex allowing the recognition of LPS by MD2 and TLR4. Data from mouse cells, however, suggest that CD14 may be dispensable for the downstream events [40–42] while data from human cells suggest that CD14 is translocated to the endosomal compartment in association with the TLR4 receptor complex [43–45], thus arguing that CD14 may be required for downstream TLR4 signaling events.

Although ligand binding and transfer by CD14 has been extensively studied by mutagenesis and epitope mapping of blocking antibodies in both human and animal models [40, 41, 46–49], the molecular mechanism behind CD14 interaction with the receptor complex remains elusive. Mechanisms for ligand-induced endocytosis of CD14 and control of endosomal trafficking of the TLR receptor complex likewise remain unclear.

Further we found that mouse complexes containing MyD88 protein are represented in two forms, containing alternatively spliced long and short isoforms of MyD88, MyD88l and MyD88s (Tables 1 and 2). The long or canonical form of MyD88 protein is a bipartite domain adaptor molecule composed of an amino-terminal death domain and a carboxyl-terminal TIR domain. MyD88l bridges interleukin-1 receptor-associated kinase 4 (IRAK4) to the TIR-domain of receptor signaling complex. The short form MyD88s lacks the region between the death domain and the TIR domain. MyD88s is also recruited to the TIR-domain of TLR4 receptor complex but it blocks NFkappaB induction because it fails to activate IRAK4 in mouse cells [50, 51]. In contrast, although human cells have been reported to express MyD88s, only TLR4 complexes involving the canonical long form of MyD88 have been observed. This difference is consistent with the lack of evidence showing that LPS-induced activity of MyD88s inhibits MyD88-mediated TLR4 pathway in human cells.

A key feature of the work described in this paper is that it involves the ***annotation*** of specific ***instances*** of physical entities: the collections of molecules in particular cells occupy a subcellular location or exhibit a function. Work now underway on development of a formal ontology for these classes and relationships will enable us to use these annotations as the basis for assertions to support automated reasoning. While the expert manual annotation process does not scale well, it does provide a large body of validated data that will provide a rigorous test of automated reasoning tools.

## Conclusion

We have described an annotation process that integrates PRO ontology terms for protein complexes with GO terms for molecular function, biological process, and cellular component. The resulting annotations are explicitly tagged to indicate their basis in experimental data or in manually verified inferences based on sequence similarity among proteins. The results highlight similarities and differences between signaling processes mediated by two members of the TLR family, TLR3 and TLR4, and among three vertebrate species, human, mouse, and chicken. This annotation strategy is readily extended to the large data sets in pathway databases like Reactome and with the continued development of ontologies and reasoning tools should allow these resources to be mined efficiently and reliably, to discover putative novel functional relationships among proteins and protein complexes and to critically assess their plausibility.

## Supporting Information

S1 Table Initiation of TLR3 and TLR4 signaling cascades. (DOCX)

S2 Table TLR3 and TLR4 complexes. (DOCX)

S3 Table TLR3 and TLR4 complex components. (DOCX)

S4 Table Annotation of species-specific functions of MD2:TLR4 complexes. (DOCX)

## References

[1] UniProt Consortium (132 collaborators) Activities at the Universal Protein Resource (UniProt). Nucleic Acids Res. 2014;42:D191–D198. 10.1093/nar/gkt1140 24253303 PMC3965022

[2] Blake JA, Bult CJ, Eppig JT, Kadin JA, Richardson JE, The Mouse Genome Database Group. The Mouse Genome Database: integration of and access to knowledge about the laboratory mouse. Nucleic Acids Res. 2014;42: D810–D817. 10.1093/nar/gkt1225 24285300 PMC3964950

[3] Croft D, Fabregat Mundo A, Haw R, Milacic M, Weiser J, Wu G, et al The Reactome Pathway Knowledgebase. Nucleic Acids Res. 2014;42: D472–D477. 10.1093/nar/gkt1102 24243840 PMC3965010

[4] Smith B, Ceusters W, Klagges B, Kohler J, Kumar A, Lomax J, et al Relations in biomedical ontologies. Genome Biol. 2005;6: R46 15892874 10.1186/gb-2005-6-5-r46 PMC1175958

[5] Ashburner M, Ball CA, Blake JA, Botstein D, Butler H, Cherry JM, et al Gene ontology: tool for the unification of biology. Nat Genet. 2000;25: 25–29. 10802651 10.1038/75556 PMC3037419

[6] Gene Ontology Consortium. Gene Ontology Consortium: going forward. Nucleic Acids Res. 2015;43: D1049–D1056 10.1093/nar/gku1179 25428369 PMC4383973

[7] Arighi CN, Liu H, Natale DA, Barker WC, Drabkin H, Blake JA, et al (2009) TGF-beta signaling proteins and the Protein Ontology. BMC Bioinformatics. 2009;10 Suppl 5: S3 10.1186/1471-2105-10-S5-S3 19426460 PMC2679403

[8] Bult C, Drabkin H, Evsikov A, Natale D, Arighi C, D’Eustachio P, et al The representation of protein complexes in the Protein Ontology (PRO). BMC Bioinformatics 2011;12: 371 10.1186/1471-2105-12-371 21929785 PMC3189193

[9] Natale DA, Arighi CN, Barker WC, Blake J, Chang TC, Hu Z, et al Framework for a protein ontology. BMC Bioinformatics. 2007;8 Suppl 9: S1 18047702 10.1186/1471-2105-8-S9-S1 PMC2217659

[10] Natale DA, Arighi CN, Blake JA, Bult CJ, Christie KR, Cowart J, et al Protein Ontology: a controlled structured network of protein entities. Nucleic Acids Res. 2014;42: D415–421. 10.1093/nar/gkt1173 24270789 PMC3964965

[11] Gillespie M, Shamovsky V, D’Eustachio P. Human and chicken TLR pathways: manual curation and computer-based orthology analysis. Mammalian Genome. 2011;22: 130–138 10.1007/s00335-010-9296-0 21052677 PMC3035812

[12] Roach JM, Racioppi L, Jones CD, Masci AM. Phylogeny of Toll-like receptor signaling: adapting the innate response. PLoS One. 2013;8:e54156 10.1371/journal.pone.0054156 23326591 PMC3543326

[13] Akira S, Uematsu S, Takeuchi O. Pathogen recognition and innate immunity. Cell. 2006;124: 783–801. 16497588 10.1016/j.cell.2006.02.015

[14] Kawai T, Akira S. The role of pattern-recognition receptors in innate immunity: update on Toll-like receptors. Nat Immunol. 2010;11: 373–384. 10.1038/ni.1863 20404851

[15] Medzhitov R. Recognition of microorganisms and activation of the immune response. Nature. 2007;449: 819–826. 17943118 10.1038/nature06246

[16] Zhang Q, Zmasek CM, Godzik A. Domain architecture evolution of pattern-recognition receptors. Immunogenetics. 2010;62: 263–272. 10.1007/s00251-010-0428-1 20195594 PMC2858798

[17] Abdelsadik A, Trad A. Toll-like receptors on the fork roads between innate and adaptive immunity. Hum Immunol. 2011;72: 1188–1193. 10.1016/j.humimm.2011.08.015 21920397

[18] Qian C, Cao X. Regulation of Toll-like receptor signaling pathways in innate immune responses. Ann N Y Acad Sci. 2013;1283: 67–74. 10.1111/j.1749-6632.2012.06786.x 23163321

[19] Sasai M, Yamamoto M. Pathogen recognition receptors: ligands and signaling pathways by Toll-like receptors. Int Rev Immunol. 2013;32: 116–133. 10.3109/08830185.2013.774391 23570313

[20] Jin MS, Lee JO. Structures of the toll-like receptor family and its ligand complexes. Immunity. 2008;29: 182–191. 10.1016/j.immuni.2008.07.007 18701082

[21] Hashimoto C, Hudson KL, Anderson KV. The Toll gene of Drosophila, required for dorsal-ventral embryonic polarity, appears to encode a transmembrane protein. Cell. 1988;52: 269–279. 2449285 10.1016/0092-8674(88)90516-8

[22] Lemaitre B, Nicolas E, Michaut L, Reichhart JM, Hoffmann JA. The dorsoventral regulatory gene cassette spatzle/Toll/cactus controls the potent antifungal response in Drosophila adults. Cell. 1996;86: 973–983. 8808632 10.1016/s0092-8674(00)80172-5

[23] Lemaitre B, Hoffmann J. The host defense of Drosophila melanogaster. Annu Rev Immunol. 2007;25: 697–743. 17201680 10.1146/annurev.immunol.25.022106.141615

[24] Matsuo A, Oshiumi H, Tsujita T, Mitani H, Kasai H, Yoshimizu M, et al Teleost TLR22 recognizes RNA duplex to induce IFN and protect cells from birnaviruses. J Immunol. 2008;181: 3474–3485. 18714020 10.4049/jimmunol.181.5.3474

[25] Roach JC, Glusman G, Rowen L, Kaur A, Purcell MK, Smith KD, et al The evolution of vertebrate Toll-like receptors. Proc Natl Acad Sci U S A. 2005;102: 9577–9582. 15976025 10.1073/pnas.0502272102 PMC1172252

[26] Masci AM, Levin M, Ruttenberg A, Cowell LG. Connecting ontologies for the representation of biological pathways. ICBO: International Conference on Biomedical Ontologies—Buffalo NY USA; 2011. Available: http://ceur-ws.org/Vol-833/paper74.pdf

[27] Hastings J, de Matos P, Dekker A, Ennis M, Harsha B, Kale N, et al The ChEBI reference database and ontology for biologically relevant chemistry: enhancements for 2013. Nucleic Acids Res. 2013;41: D456–D463. 10.1093/nar/gks1146 23180789 PMC3531142

[28] Arighi C. PRO Ontology Manual Curation Guideline https://pir17.georgetown.edu/confluence/display/PROWIKI/PRO+Ontology+Manual+Curation+Guideline. Accessed 11 March 2015.

[29] Smith B, Ashburner M, Rosse C, Bard J, Bug W, Ceusters W, et al (2007) The OBO Foundry: coordinated evolution of ontologies to support biomedical data integration. Nat Biotechnol. 2007;25: 1251–1255. 17989687 10.1038/nbt1346 PMC2814061

[30] OBO Relation Ontology http://www.ontobee.org/browser/index.php?o=RO. Accessed 11 March 2015.

[31] Jupe S, Jassal B, Williams M, Wu G. A controlled vocabulary for pathway entities and events. Database. 2008;bau060. 10.1093/database/bau060 PMC4064568 24951798

[32] PRO terms and annotations. ftp://ftp.pir.georgetown.edu/databases/ontology/pro_obo/TLR/. Accessed 11 March 2015.

[33] PAF Guidelines ftp://ftp.pir.georgetown.edu/databases/ontology/pro_obo/PAF_guidelines.pdf. Accessed 11 March 2015.

[34] Keestra AM, van Putten JPM. Unique properties of the chicken TLR4/MD-2 complex: selective lipopolysaccharide activation of the MyD88-dependent pathway. J Immunol. 2008;181: 4354–4362. 18768894 10.4049/jimmunol.181.6.4354

[35] Esnault E, Bonsergent C, Larcher T, Bed'hom B, Vautherot JF, Delaleu B, et al (2011) A novel chicken lung epithelial cell line: characterization and response to low pathogenicity avian influenza virus. Virus Res. 2011;159: 32–42. 10.1016/j.virusres.2011.04.022 21557972

[36] Vaure C, Liu Y. A comparative review of toll-like receptor 4 expression and functionality in different animal species. Front Immunol. 2014;5: 316 10.3389/fimmu.2014.00316 25071777 PMC4090903

[37] Pålsson-McDermott EM, O'Neill LA. (2004) Signal transduction by the lipopolysaccharide receptor, Toll-like receptor-4. Immunology. 2004;113: 153–162. 15379975 10.1111/j.1365-2567.2004.01976.x PMC1782563

[38] Wu Z, Rothwell L, Hu T, Kaiser P. Chicken CD14, unlike mammalian CD14, is trans-membrane rather than GPI-anchored. Dev Comp Immunol. 2009;33: 97–104. 10.1016/j.dci.2008.07.008 18761368

[39] Haddadi S, Kim DS, Jasmine H, van der Meer F, Czub M, Abdul-Careem MF. Induction of Toll-like receptor 4 signaling in avian macrophages inhibits infectious laryngotracheitis virus replication in a nitric oxide dependent way. Vet Immunol Immunopathol. 2013;155: 270–275. 10.1016/j.vetimm.2013.08.005 24034933

[40] da Silva Correia J, Soldau K, Christen U, Tobias PS, Ulevitch RJ. Lipopolysaccharide is in close proximity to each of the proteins in its membrane receptor complex. transfer from CD14 to TLR4 and MD-2. J Biol Chem. 2001;276: 21129–21135. 11274165 10.1074/jbc.M009164200

[41] Akashi S, Saitoh S, Wakabayashi Y, Kikuchi T, Takamura N, Nagai Y, et al Lipopolysaccharide interaction with cell surface Toll-like receptor 4-MD-2: higher affinity than that with MD-2 or CD14. J Exp Med. 2003;198: 1035–1042. 14517279 10.1084/jem.20031076 PMC2194215

[42] Park BS, Song DH, Kim HM, Choi BS, Lee H, Lee JO. The structural basis of lipopolysaccharide recognition by the TLR4-MD-2 complex. Nature. 2009;458: 1191–1195. 10.1038/nature07830 19252480

[43] Husebye H, Halaas Ø, Stenmark H, Tunheim G, Sandanger Ø, Bogen B, et al Endocytic pathways regulate Toll-like receptor 4 signaling and link innate and adaptive immunity. EMBO J. 2006;25:683–692. 16467847 10.1038/sj.emboj.7600991 PMC1383569

[44] Zanoni I, Ostuni R, Marek LR, Barresi S, Barbalat R, Barton GM, et al (2011) CD14 controls the LPS-induced endocytosis of Toll-like receptor 4. Cell. 2011;147: 868–80. 10.1016/j.cell.2011.09.051 22078883 PMC3217211

[45] Roy S, Karmakar M, Pearlman E. CD14 mediates Toll-like receptor 4 (TLR4) endocytosis and spleen tyrosine kinase (Syk) and interferon regulatory transcription factor 3 (IRF3) activation in epithelial cells and impairs neutrophil infiltration and Pseudomonas aeruginosa killing in vivo. J Biol Chem. 2014;289: 1174–82. 10.1074/jbc.M113.523167 24275652 PMC3887184

[46] Jiang Q, Akashi S, Miyake K, Petty HR. Lipopolysaccharide induces physical proximity between CD14 and toll-like receptor 4 (TLR4) prior to nuclear translocation of NF-kappa B. J Immunol. 2000;165:3541–3544. 11034352 10.4049/jimmunol.165.7.3541

[47] Teghanemt A, Prohinar P, Gioannini TL, Weiss JP. Transfer of monomeric endotoxin from MD-2 to CD14: characterization and functional consequences. J Biol Chem. 2007;282: 36250–36256. 17934216 10.1074/jbc.M705995200

[48] Tsukamoto H, Fukudome K, Takao S, Tsuneyoshi N, Kimoto M. Lipopolysaccharide-binding protein-mediated Toll-like receptor 4 dimerization enables rapid signal transduction against lipopolysaccharide stimulation on membrane-associated CD14-expressing cells. Int Immunol. 2010;22: 271–80. 10.1093/intimm/dxq005 20133493

[49] Kim D, Kim JY. Anti-CD14 antibody reduces LPS responsiveness via TLR4 internalization in human monocytes. Mol Immunol. 2014;57: 210–215. 10.1016/j.molimm.2013.09.009 24172224

[50] Janssens S, Burns K, Tschopp J, Beyaert R. Regulation of interleukin-1- and lipopolysaccharide-induced NF-kappaB activation by alternative splicing of MyD88. Curr Biol. 2002;12: 467–471. 11909531 10.1016/s0960-9822(02)00712-1

[51] Burns K, Janssens S, Brissoni B, Olivos N, Beyaert R, Tschopp J. Inhibition of interleukin 1 receptor/Toll-like receptor signaling through the alternatively spliced, short form of MyD88 is due to its failure to recruit IRAK-4. J Exp Med. 2003;197: 263–268. 12538665 10.1084/jem.20021790 PMC2193806

[52] Kim HM, Park BS, Kim JI, Kim SE, Lee J, Oh SC, et al Crystal structure of the TLR4-MD-2 complex with bound endotoxin antagonist Eritoran. Cell. 2007;30: 906–917. 10.1016/j.cell.2007.08.002 17803912

