# Correction: Toll-Like Receptor Signaling in Vertebrates: Testing the Integration of Protein, Complex, and Pathway Data in the Protein Ontology Framework

**DOI:** 10.1371/journal.pone.0131148

**Published:** 2015-06-18

**Authors:** Cecilia Arighi, Veronica Shamovsky, Anna Maria Masci, Alan Ruttenberg, Barry Smith, Darren A. Natale, Cathy Wu, Peter D’Eustachio

A grant number is missing in the Funding section. The correct funding information is as follows: The project is supported by the National Institutes of Health grants 5R01GM080646-09 and HHSN272201000053C (www.nih.gov). The funder had no role in study design, data collection and analysis, decision to publish, or preparation of the manuscript.
