# Supplementary material for: Toll-Like Receptor Signaling in Vertebrates: Testing the Integration of Protein, Complex, and Pathway Data in the Protein Ontology Framework
Source: PLoS One. 2015 Apr 20;10(4):e0122978. doi: 10.1371/journal.pone.0122978 (PMC4404318; doi:10.1371/journal.pone.0122978)
Supplement: S1 Table — (DOCX) [file pone.0122978.s001.docx]

**S1 Table. Initiation of TLR3 and TLR4 signaling cascades***

**Reaction Entity identifiers Entity copy number**input and output entities

**TLR3 PATHWAY**

**Human**:
**Reaction:** [**Viral dsRNA binds the Toll-Like Receptor 3 (TLR3)**](http://www.reactome.org/cgi-bin/control_panel_st_id?ST_ID=REACT_6753.2) [[REACT_6753](http://www.reactome.org/cgi-bin/eventbrowser_st_id?ST_ID=REACT_6753)]
Input: mature TLR3 (signal peptide removed) [PR:000037305](http://pir.georgetown.edu/cgi-bin/pro/entry_pro?id=PR:000037305) 2
 viral dslRNA [CHEBI:67208](http://www.ebi.ac.uk/chebi/advancedSearchFT.do?searchString=67208&queryBean.stars=2) 1
Output: TLR3:viral dsRNA complex [PR:000037303](http://pir.georgetown.edu/cgi-bin/pro/entry_upro?id=PR:000037303) 1 of complex
**Reaction:** [**Viral dsRNA:TLR3 recruits TRIF (TICAM1)**](http://www.reactome.org/cgi-bin/control_panel_st_id?ST_ID=REACT_6919.2) [[REACT_6919](http://www.reactome.org/cgi-bin/eventbrowser_st_id?ST_ID=REACT_6919)]
Input: TLR3:dsViralRNA complex [PR:000037303](http://pir.georgetown.edu/cgi-bin/pro/entry_upro?id=PR:000037303) 1
 TICAM1 [PR:Q8IUC6](http://pir.georgetown.edu/cgi-bin/pro/entry_upro?id=PR:Q8IUC6) 2
Output TICAM1:TLR3:viral dsRNA complex [PR:000037307](http://pir.georgetown.edu/cgi-bin/pro/entry_upro?id=PR:000037307) 1 of complex

**Chicken**:
**Reaction:** [**viral dsRNA binds the toll-like receptor 3 (TLR3)**](http://www.reactome.org/cgi-bin/control_panel_st_id?ST_ID=REACT_24923.1) [[REACT_24923](http://www.reactome.org/cgi-bin/eventbrowser_st_id?ST_ID=REACT_24923)]
Input: TLR3 [PR:Q0PQ88](http://pir.georgetown.edu/cgi-bin/pro/entry_upro?id=PR:Q0PQ88) 2
 viral dsRNA [CHEBI:67208](http://www.ebi.ac.uk/chebi/advancedSearchFT.do?searchString=67208&queryBean.stars=2) 1
Output: TLR3:viral dslRNA complex [PR:000037461](http://pir.georgetown.edu/cgi-bin/pro/entry_pro?id=PR:000037461) 1 of complex
**Reaction:** [**viral dsRNA:TRL3 recruits TICAM1**](http://www.reactome.org/cgi-bin/control_panel_st_id?ST_ID=REACT_25180.2) [[REACT_](http://www.reactome.org/cgi-bin/control_panel_st_id?ST_ID=REACT_25180.2)[25180](http://www.reactome.org/cgi-bin/eventbrowser_st_id?ST_ID=REACT_25180)]
Input: TLR3:viral dsRNA complex [PR:000037461](http://pir.georgetown.edu/cgi-bin/pro/entry_pro?id=PR:000037461) 1 of complex
 TICAM1 [PR:A0FKC7](http://pir.georgetown.edu/cgi-bin/pro/entry_upro?id=PR:A0FKC7) 2
Output: TICAM1:TLR3:viral dsRNA complex [PR: 000037462](http://pir.georgetown.edu/cgi-bin/pro/entry_pro?id=PR:000037462) 1 of complex

**Mouse**:
**Reaction: viral dsRNA binds to the toll like receptor 3 (Tlr3)**
Input: TLR3 [PR:Q99MB1](http://pir.georgetown.edu/cgi-bin/pro/entry_upro?id=PR:Q99MB1) 2
 viral dsRNA [CHEBI:67208](http://www.ebi.ac.uk/chebi/searchId.do?chebiId=CHEBI:67208) 1
Output: TLR3:viral dsRNA complex [PR:000037304](http://pir.georgetown.edu/cgi-bin/pro/entry_pro?id=PR:000037304) 1 of complex
**Reaction: viral dsRNA:Tlr3 recruits Ticam1**
Input: TLR3:viral dsRNA complex [PR:000037304](http://pir.georgetown.edu/cgi-bin/pro/entry_pro?id=PR:000037304) 1 of complex
 TICAM1 [PR:Q80UF7](http://pir.georgetown.edu/cgi-bin/pro/entry_pro?id=PR:Q80UF7) 2
Output: TICAM1:TLR3:viral dsRNA complex [PR:000037308](http://pir.georgetown.edu/cgi-bin/pro/entry_pro?id=PR:000037308) 1 of complex

**TLR4 PATHWAY**

**Human**:
**Reaction:** [**Transfer of LPS onto TLR4**](http://www.reactome.org/cgi-bin/control_panel_st_id?ST_ID=REACT_6795.2) [[REACT_6795](http://www.reactome.org/cgi-bin/eventbrowser_st_id?ST_ID=REACT_6795)]
Input modified TLR4:modified MD2 (LY96) [PR:000036004](http://pir.georgetown.edu/cgi-bin/pro/entry_pro?id=PR:000036004) 2 of complex
 TLR4 [PR:O00206](http://pir.georgetown.edu/cgi-bin/pro/entry_upro?id=PR:O00206) 1
 LY96 (MD2) [PR:Q9Y6Y9](http://pir.georgetown.edu/cgi-bin/pro/entry_upro?id=PR:Q9Y6Y9) 1
 LPS:CD14 (modified or unmodified) [PR:000037464](http://pir.georgetown.edu/cgi-bin/pro/entry_pro?id=PR:000037464), [PR:000037463](http://pir.georgetown.edu/cgi-bin/pro/entry_pro?id=PR:000037463) 2 of complex
 CD14 [PR:P08571](http://pir.georgetown.edu/cgi-bin/pro/entry_upro?id=PR:P08571) 1
 LPS [ChEBI 16412](http://www.ebi.ac.uk/chebi/searchId.do?chebiId=CHEBI:16412) 1
Output: 2 TLR4:LY96:LPS:CD14 complex [PR:000025773](http://pir.georgetown.edu/cgi-bin/pro/entry_pro?id=PR:000025773) 1 of complex
**Reaction:** [**Activated TLR2/4 interacts with MAL(TIRAP)**](http://www.reactome.org/cgi-bin/control_panel_st_id?ST_ID=REACT_121383.4) [[REACT_121383](http://www.reactome.org/cgi-bin/control_panel_st_id?ST_ID=REACT_121383.3)]
Input: TLR4:LY96:LPS:CD14 complex [PR:000025773](http://pir.georgetown.edu/cgi-bin/pro/entry_pro?id=PR:000025773) 2 of complex
 TIRAP:PI_4,5_P2 complex [PR:000036014](http://pir.georgetown.edu/cgi-bin/pro/entry_pro?id=PR:000036014) 2 of complex
 TIRAP (MAL) [PR:P58753](http://pir.georgetown.edu/cgi-bin/pro/entry_upro?id=PR:P58753) 1
 PI_4,5_P2 [ChEBI:18348](http://www.ebi.ac.uk/chebi/searchId.do?chebiId=CHEBI:18348) 1
Output: TLR4:LY96:LPS:TIRAP: PI_4,5_P2 [PR:000036135](http://pir.georgetown.edu/cgi-bin/pro/entry_pro?id=PR:000036135) 2 of complex
**Reaction:** [**TLR4:MD2:LPS:CD14 recruits TRAM (TICAM2)**](http://www.reactome.org/cgi-bin/control_panel_st_id?ST_ID=REACT_6808.2) [[REACT_6808](http://www.reactome.org/cgi-bin/control_panel_st_id?ST_ID=REACT_6808.8)]
Input: TLR4:LY96:LPS:CD14 complex [PR:000025773](http://pir.georgetown.edu/cgi-bin/pro/entry_pro?id=PR:000025773) 2 of complex
 TICAM2 [PR:Q86XR7](http://pir.georgetown.edu/cgi-bin/pro/entry_upro?id=PR:Q86XR7) 2
Output: TRAM:TLR4:MD2:LPS:CD14 [PR:000028678](http://pir.georgetown.edu/cgi-bin/pro/entry_pro?id=PR:000028678) 2 of complex

**Chicken** :
**Reaction:** [**LPS binding to Toll like receptor 4 results in dimerization of the TLR4 associated with MD2**](http://www.reactome.org/cgi-bin/control_panel_st_id?ST_ID=REACT_25094.2) [[REACT_25094](http://www.reactome.org/cgi-bin/control_panel_st_id?ST_ID=REACT_25094.1)]
Input: TLR4:MD2 complex [PR:000037473](http://pir.georgetown.edu/cgi-bin/pro/entry_pro?id=PR:000037473) 2 of complex
 TLR4 [PR:Q7ZTG5](http://pir.georgetown.edu/cgi-bin/pro/entry_upro?id=PR:Q7ZTG5) 1
 LY96 (MD2) [PR:R4GJR4](http://pir.georgetown.edu/cgi-bin/pro/entry_upro?id=PR:R4GJR4) 1
 CD14:LPS complex [PR:000037466](http://pir.georgetown.edu/cgi-bin/pro/entry_pro?id=PR:000037466) 1 of complex
 CD14 [PR:B0BL87](http://pir.georgetown.edu/cgi-bin/pro/entry_upro?id=PR:B0BL87) 1
 LPS [ChEBI 16412](http://www.ebi.ac.uk/chebi/searchId.do?chebiId=CHEBI:16412) 1
Output: TLR4:MD2:LPS complex [PR:000037474](http://pir.georgetown.edu/cgi-bin/pro/entry_pro?id=PR:000037474) 2 of complex
 CD14 [PR:B0BL87](http://pir.georgetown.edu/cgi-bin/pro/entry_upro?id=PR:B0BL87) 1
**Reaction: Activated TLR** [**homo**](http://www.reactome.org/cgi-bin/control_panel_st_id?ST_ID=REACT_25280.3)**- or heterodimer recruits adaptors TIRAP(or MAL) and MyD88** [[REACT_25280](http://www.reactome.org/cgi-bin/control_panel_st_id?ST_ID=REACT_25280.2)]
Input: TLR4:MD2:LPS complex [PR:000037474](http://pir.georgetown.edu/cgi-bin/pro/entry_pro?id=PR:000037474) 2 of complex
 MyD88 [PR:A5HNF6](http://pir.georgetown.edu/cgi-bin/pro/entry_upro?id=PR:A5HNF6) 2
 TIRAP [PR:Q4U127](http://pir.georgetown.edu/cgi-bin/pro/entry_upro?id=PR:Q4U127) 2
Output: TLR4 dimer: TIRAP:MyD88 complex [PR:000037475](http://pir.georgetown.edu/cgi-bin/pro/entry_pro?id=PR:000037475) 1

**Mouse**:
**Reaction: Transfer of LPS onto Tlr4**
Input: modified TLR4:modified MD2 (LY96) [PR:000036005](http://pir.georgetown.edu/cgi-bin/pro/entry_pro?id=PR:000036005) 2 of complex
 LY96 (MD2) [PR:000027171](http://pir.georgetown.edu/cgi-bin/pro/entry_pro?id=PR:000027171) 1
 LPS:CD14 (modified or unmodified) [PR:000037467](http://pir.georgetown.edu/cgi-bin/pro/entry_pro?id=PR:000037467), [PR:000037468](http://pir.georgetown.edu/cgi-bin/pro/entry_pro?id=PR:000037468) 1 of each
 CD14  [PR:P10810](http://pir.georgetown.edu/cgi-bin/pro/entry_pro?id=PR:P10810) 1
 LPS [CHEBI:16412](http://www.ebi.ac.uk/chebi/searchId.do?chebiId=CHEBI:16412) 1
Output: TLR4:LY96:LPS complex [PR:000036077](http://pir.georgetown.edu/cgi-bin/pro/entry_pro?id=PR:000036077) 1 of complex
 CD14  [PR:P10810](http://pir.georgetown.edu/cgi-bin/pro/entry_pro?id=PR:P10810) 1
**Reaction: TIRAP:TLR4:LY96:LPS complex interacts with MyD88**
Input: TIRAP:TLR4:LY96:LPS [PR:000036079](http://pir.georgetown.edu/cgi-bin/pro/entry_pro?id=PR:000036079) 1 of complex
 MyD88 [PR:000025766](http://pir.georgetown.edu/cgi-bin/pro/entry_pro?id=PR:000025766) 1
Output: MyD88:Mal:activated TLR4 receptor [PR:000027175](http://pir.georgetown.edu/cgi-bin/pro/entry_pro?id=PR:000027175) 1 of complex
**Reaction: Activated Tlr4 interacts with Tirap (Mal)**
Input TLR4:LY96:LPS complex [PR:000036077](http://pir.georgetown.edu/cgi-bin/pro/entry_pro?id=PR:000036077) 1 of complex
 Tirap:pip2 complex [PR:000036007](http://pir.georgetown.edu/cgi-bin/pro/entry_pro?id=PR:000036007) 1 of complex
Output TIRAP:PIP2:MD2:LPS:TLR4 complex [PR:000036079](http://pir.georgetown.edu/cgi-bin/pro/entry_pro?id=PR:000036079) 1 of complex

* Signaling via TLR3 and TLR4 is initiated in two reactions: the binding of a foreign ligand to host TLR and the interaction of the resulting ligand:receptor complex with host accessory proteins. For each reaction in human, mouse, and chicken, the input physical entities (proteins, protein complexes, and nonprotein molecules) are shown together with the number of copies of each, and the output complex that results is shown. Proteins and complexes are named following PRO conventions; nonprotein entities are named following ChEBI conventions. The human and chicken versions of all of these reactions have been annotated in Reactome and the Reactome name and stable identifier for each reaction, e.g., “Viral dsRNA binds the Toll-Like Receptor 3 (TLR3) [REACT_6753]”, are shown.
