# Supplementary material for: Toll-Like Receptor Signaling in Vertebrates: Testing the Integration of Protein, Complex, and Pathway Data in the Protein Ontology Framework
Source: PLoS One. 2015 Apr 20;10(4):e0122978. doi: 10.1371/journal.pone.0122978 (PMC4404318; doi:10.1371/journal.pone.0122978)
Supplement: S2 Table — (DOCX) [file pone.0122978.s002.docx]

**S2 Table. TLR3 and TLR4 complexes**

| **parent PRO ID** | **name** | **human PRO ID** | **human Reactome ID** | **mouse PRO ID** |
| --- | --- | --- | --- | --- |
| [PR:000037302](http://pir.georgetown.edu/cgi-bin/pro/entry_pro?id=PR:000037302) | viral dsRNA:TLR3 complex | [PR:000037303](http://pir.georgetown.edu/cgi-bin/pro/entry_upro?id=PR:000037303) | [REACT_7159](http://www.reactome.org/cgi-bin/eventbrowser_st_id?ST_ID=REACT_7159) | [PR:000037304](http://pir.georgetown.edu/cgi-bin/pro/entry_pro?id=PR:000037304) |
| [PR:000037306](http://pir.georgetown.edu/cgi-bin/pro/entry_pro?id=PR:000037306) | ticam1:viral dsRNA:TLR3 complex | [PR:000037307](http://pir.georgetown.edu/cgi-bin/pro/entry_upro?id=PR:000037307) | [REACT_7381](http://www.reactome.org/cgi-bin/eventbrowser_st_id?ST_ID=REACT_7381) | [PR:000037308](http://pir.georgetown.edu/cgi-bin/pro/entry_pro?id=PR:000037308) |
| [PR:000037309](http://pir.georgetown.edu/cgi-bin/pro/entry_pro?id=PR:000037309) | traf3:ticam1:activated TLR3 complex | [PR:000037310](http://pir.georgetown.edu/cgi-bin/pro/entry_pro?id=PR:000037310) | [REACT_124037](http://www.reactome.org/cgi-bin/eventbrowser_st_id?ST_ID=REACT_124037) | [PR:000037311](http://pir.georgetown.edu/cgi-bin/pro/entry_pro?id=PR:000037311) |
| [PR:000037343](http://pir.georgetown.edu/cgi-bin/pro/entry_pro?id=PR:000037343) | traf6:ticam1:activated TLR3 complex | [PR:000037344](http://pir.georgetown.edu/cgi-bin/pro/entry_pro?id=PR:000037344) | [REACT_25948](http://www.reactome.org/cgi-bin/eventbrowser_st_id?ST_ID=REACT_25948) | [PR:000037471](http://pir.georgetown.edu/cgi-bin/pro/entry_pro?id=PR:000037471) |
| [PR:000036003](http://pir.georgetown.edu/cgi-bin/pro/entry_pro?id=PR:000036003) | MD2:TLR4 complex | [PR:000036004](http://pir.georgetown.edu/cgi-bin/pro/entry_pro?id=PR:000036004) | [REACT_7105](http://www.reactome.org/cgi-bin/eventbrowser_st_id?ST_ID=REACT_7105) | [PR:000036005](pr:000036005) |
| [PR:000036076](http://pir.georgetown.edu/cgi-bin/pro/entry_pro?id=PR:000036076) | MD2:LPS:TLR4 complex | none | none | [PR:000036077](http://pir.georgetown.edu/cgi-bin/pro/entry_pro?id=PR:000036077) |
| [PR:000025497](http://pir.georgetown.edu/cgi-bin/pro/entry_pro?id=PR:000025497) | lipopolysaccharide receptor complex 3 | [PR:000025773](http://pir.georgetown.edu/cgi-bin/pro/entry_pro?id=PR:000025773) | [REACT_124771](http://www.reactome.org/cgi-bin/eventbrowser_st_id?ST_ID=REACT_124771) | [PR:000037476](http://pir.georgetown.edu/cgi-bin/pro/entry_pro?id=PR:000037476) |
| [PR:000025498](http://pir.georgetown.edu/cgi-bin/pro/entry_pro?id=PR:000025498) | lipopolysaccharide receptor complex 4 | [PR:000037479](http://pir.georgetown.edu/cgi-bin/pro/entry_pro?id=PR:000037479) | [REACT_124771](http://www.reactome.org/cgi-bin/eventbrowser_st_id?ST_ID=REACT_124771) | none |
| [PR:000027202](http://pir.georgetown.edu/cgi-bin/pro/entry_pro?id=PR:000027202) | ticam2:activated TLR4 complex | [PR:000028678](http://pir.georgetown.edu/cgi-bin/pro/entry_pro?id=PR:000028678) | [REACT_7083](http://www.reactome.org/cgi-bin/eventbrowser_st_id?ST_ID=REACT_7083) | [PR:000027204](http://pir.georgetown.edu/cgi-bin/pro/entry_pro?id=PR:000027204) |
| [PR:000027205](http://pir.georgetown.edu/cgi-bin/pro/entry_pro?id=PR:000027205) | ticam1:ticam2:activated TLR4 complex | [PR:000027208](http://pir.georgetown.edu/cgi-bin/pro/entry_pro?id=PR:000027208) | [REACT_7861](http://www.reactome.org/cgi-bin/eventbrowser_st_id?ST_ID=REACT_7861) | [PR:000027207](http://pir.georgetown.edu/cgi-bin/pro/entry_pro?id=PR:000027207) |
| [PR:000028681](http://pir.georgetown.edu/cgi-bin/pro/entry_pro?id=PR:000028681) | traf3:ticam1:ticam2:activated TLR4 complex | [PR:000036022](http://pir.georgetown.edu/cgi-bin/pro/entry_pro?id=PR:000036022) | [REACT_124037](http://www.reactome.org/cgi-bin/eventbrowser_st_id?ST_ID=REACT_124037) | [PR:000028683](http://pir.georgetown.edu/cgi-bin/pro/entry_pro?id=PR:000028683) |
| [PR:000028679](http://pir.georgetown.edu/cgi-bin/pro/entry_pro?id=PR:000028679) | traf6:ticam1:ticam2:activated TLR4 complex | PR:000028680 | [REACT_25948](http://www.reactome.org/cgi-bin/eventbrowser_st_id?ST_ID=REACT_25948) | [PR:000035710](http://pir.georgetown.edu/cgi-bin/pro/entry_pro?id=PR:000035710) |
| [PR:000036078](http://pir.georgetown.edu/cgi-bin/pro/entry_pro?id=PR:000036078) | TIRAP:PIP2:activated TLR4 complex | [PR:000036135](http://pir.georgetown.edu/cgi-bin/pro/entry_pro?id=PR:000036135) | [REACT_152404](http://www.reactome.org/cgi-bin/eventbrowser_st_id?ST_ID=REACT_152404) | [PR:000027196](http://pir.georgetown.edu/cgi-bin/pro/entry_pro?id=PR:000036079) |
| [PR:000037472](http://pir.georgetown.edu/cgi-bin/pro/entry_pro?id=PR:000037472) | TIRAP:PIP2:BTK:activated TLR4 complex | [PR:000037447](http://pir.georgetown.edu/cgi-bin/pro/entry_pro?id=PR:000037447&retrieve.x=0&retrieve.y=0) | [REACT_124673](http://www.reactome.org/cgi-bin/eventbrowser_st_id?ST_ID=REACT_124673) | [PR:000037477](http://pir.georgetown.edu/cgi-bin/pro/entry_pro?id=PR:000037477)+ |
| [PR:000037472](http://pir.georgetown.edu/cgi-bin/pro/entry_pro?id=PR:000037472) | pTIRAP:PIP2:BTK:activated TLR4 complex | [PR:000037448](http://pir.georgetown.edu/cgi-bin/pro/entry_pro?id=PR:000037448&retrieve.x=0&retrieve.y=0) | [REACT_125282](http://www.reactome.org/cgi-bin/eventbrowser_st_id?ST_ID=REACT_125282) | [PR:000027217](http://pir.georgetown.edu/cgi-bin/pro/entry_pro?id=PR:000027217) |
| [PR:000037488](http://pir.georgetown.edu/cgi-bin/pro/entry_pro?id=PR:000037488) | MyD88:TIRAP:PIP2:BTK:activated TLR4 complex | [PR:000037446](http://pir.georgetown.edu/cgi-bin/pro/entry_pro?id=PR:000037446&retrieve.x=0&retrieve.y=0) | [REACT_7694](http://www.reactome.org/cgi-bin/eventbrowser_st_id?ST_ID=REACT_7694) | [PR:000037481](http://pir.georgetown.edu/cgi-bin/pro/entry_pro?id=PR:000037481)+ |
| [PR:000025784](http://pir.georgetown.edu/cgi-bin/pro/entry_pro?id=PR:000025784) | MyD88:Mal:activated TLR4 receptor | none | none | [PR:000027174](http://pir.georgetown.edu/cgi-bin/pro/entry_pro?id=PR:000027174) |

+ All PRO annotations are based on experimental evidence (Evidence code ontology ECO:0000269) except ones marked with asterisks, which are based on reconstruction of a biological system (ECO:0000088)

For each complex involved in the initial steps of TLR3 or TLR4 signaling (Table 1), the PRO identifier of its species-agnostic form (parent PRO ID) is listed, together with its PRO name and the PRO identifiers of its human and mouse forms and the Reactome identifier of its human form. In this version of the table published in the paper, hyperlinks have been embedded in all database identifiers, allowing direct access to these resources. The two tables are otherwise identical.
