# Supplementary material for: Toll-Like Receptor Signaling in Vertebrates: Testing the Integration of Protein, Complex, and Pathway Data in the Protein Ontology Framework
Source: PLoS One. 2015 Apr 20;10(4):e0122978. doi: 10.1371/journal.pone.0122978 (PMC4404318; doi:10.1371/journal.pone.0122978)
Supplement: S3 Table — (DOCX) [file pone.0122978.s003.docx]

**S3 Table. TLR3 and TLR4 complex components**

| **parent PRO ID** | **Name** | **UniProtKB (human) or ChEBI** | **PRO ID human** | **UniProtKB (mouse) or ChEBI** | **PRO ID mouse** |
| --- | --- | --- | --- | --- | --- |
| none | dsRNA | [CHEBI:67208](http://www.ebi.ac.uk/chebi/searchId.do?chebiId=CHEBI:67208) | none | [CHEBI:67208](http://www.ebi.ac.uk/chebi/searchId.do?chebiId=CHEBI:67208) | None |
| none | lipopolysaccharide | [CHEBI:16412](http://www.ebi.ac.uk/chebi/searchId.do?chebiId=CHEBI:16412) | none | [CHEBI:16412](http://www.ebi.ac.uk/chebi/searchId.do?chebiId=CHEBI:16412) | None |
| none | 1-phosphatidyl-1D-myo-inositol 4,5-bisphosphate | [CHEBI:18348](http://www.ebi.ac.uk/chebi/searchId.do?chebiId=CHEBI:18348) | none | [CHEBI:18348](http://www.ebi.ac.uk/chebi/searchId.do?chebiId=CHEBI:18348) | None |
| [PR:000025492](http://pir.georgetown.edu/cgi-bin/pro/entry_pro?id=PR:000025492) | Toll-like receptor 4 isoform 1, signal peptide removed glycosylated 1 | [O00206-1](http://www.uniprot.org/uniprot/O00206-1) | [PR:000025787](http://pir.georgetown.edu/cgi-bin/pro/entry_pro?id=PR:000025787) | [Q9QUK6-1](http://www.uniprot.org/uniprot/Q9QUK6-1) | [PR:000027172](http://pir.georgetown.edu/cgi-bin/pro/entry_pro?id=PR:000027172) |
| [PR:000018357](http://pir.georgetown.edu/cgi-bin/pro/entry_pro?id=PR:000018357) | Toll-like receptor 3, signal peptide removed form | [O15455](http://www.uniprot.org/uniprot/O15455) | [PR:000037305](http://pir.georgetown.edu/cgi-bin/pro/entry_pro?id=PR:000037305) | [Q99MB1](http://www.uniprot.org/uniprot/Q99MB1) | [PR:Q99MB1](http://pir.georgetown.edu/cgi-bin/pro/entry_pro?id=PR:Q99MB1) |
| [PR:000003299](http://pir.georgetown.edu/cgi-bin/pro/entry_pro?id=PR:000003299) | lymphocyte antigen 96 isoform 1, signal peptide removed, glycosylated 1 | [Q9Y6Y9-1](http://www.uniprot.org/uniprot/Q9Y6Y9-1) | [PR:000025786](http://pir.georgetown.edu/cgi-bin/pro/entry_pro?id=PR:000025786) | [Q9JHF9-1](http://www.uniprot.org/uniprot/Q9JHF9-1) | [PR:000027171](http://pir.georgetown.edu/cgi-bin/pro/entry_pro?id=PR:000027171) |
| [PR:000001749](http://pir.georgetown.edu/cgi-bin/pro/entry_upro?id=PR:000001749) | TIR domain-containing adapter molecule 1 (TICAM1) | [Q8IUC6](http://www.uniprot.org/uniprot/Q8IUC6) | [PR:Q8IUC6](http://pir.georgetown.edu/cgi-bin/pro/entry_pro?id=PR:Q8IUC6) | [Q80UF7](http://www.uniprot.org/uniprot/Q80UF7) | [PR:Q80UF7](http://pir.georgetown.edu/cgi-bin/pro/entry_pro?id=PR:Q80UF7) |
| [PR:000002289](http://pir.georgetown.edu/cgi-bin/pro/entry_upro?id=PR:000002289) | TNF receptor-associated factor 3 (TRAF3) | [Q13114](http://www.uniprot.org/uniprot/Q13114) | [PR:Q13114](http://pir.georgetown.edu/cgi-bin/pro/entry_pro?id=PR:Q13114) | [Q60803](http://www.uniprot.org/uniprot/Q60803) | [PR:Q60803](http://pir.georgetown.edu/cgi-bin/pro/entry_pro?id=PR:Q60803) |
| [PR:000002292](http://pir.georgetown.edu/cgi-bin/pro/entry_upro?id=PR:000002292) | TNF receptor-associated factor 6 | [Q9Y4K3](http://www.uniprot.org/uniprot/Q9Y4K3) | [PR:Q9Y4K3](http://pir.georgetown.edu/cgi-bin/pro/entry_pro?id=PR:Q9Y4K3) | [P70196](http://www.uniprot.org/uniprot/P70196) | [PR:P70196](http://pir.georgetown.edu/cgi-bin/pro/entry_pro?id=PR:P70196) |
| [PR:000001750](http://pir.georgetown.edu/cgi-bin/pro/entry_upro?id=PR:000001750) | TIR domain-containing adapter molecule 2 | [Q86XR7](http://www.uniprot.org/uniprot/Q86XR7) | [PR:Q86XR7](http://pir.georgetown.edu/cgi-bin/pro/entry_pro?id=PR:Q86XR7) | [Q8BJQ4](http://www.uniprot.org/uniprot/Q8BJQ4) | [PR:Q8BJQ4](http://pir.georgetown.edu/cgi-bin/pro/entry_pro?id=PR:Q8BJQ4) |
| [PR:000001740](http://pir.georgetown.edu/cgi-bin/pro/entry_pro?id=PR:000001740) | myeloid differentiation primary response protein MyD88 | [Q99836](http://www.uniprot.org/uniprot/Q99836) | [PR:Q99836](http://pir.georgetown.edu/cgi-bin/pro/entry_pro?id=PR:Q99836) | [P22366-1](http://www.uniprot.org/uniprot/P22366-1) | [PR:000025766](http://pir.georgetown.edu/cgi-bin/pro/entry_pro?id=PR:000025766) |
| [PR:000024846](http://pir.georgetown.edu/cgi-bin/pro/entry_pro?id=PR:000024846) | myeloid differentiation primary response protein MyD88 isoform 2 | none | none | [P22366-2](http://www.uniprot.org/uniprot/P22366-2) | [PR:000025767](http://pir.georgetown.edu/cgi-bin/pro/entry_pro?id=PR:000025767) |
| [PR:000001751](http://pir.georgetown.edu/cgi-bin/pro/entry_upro?id=PR:000001751) | Toll/interleukin-1 receptor domain-containing adapter protein | [P58753](http://www.uniprot.org/uniprot/P58753) | [PR:P58753](http://pir.georgetown.edu/cgi-bin/pro/entry_pro?id=PR:P58753) | [Q99JY1](http://www.uniprot.org/uniprot/Q99JY1) | [PR:Q99JY1](http://pir.georgetown.edu/cgi-bin/pro/entry_pro?id=PR:Q99JY1) |
| [PR:000027213](http://pir.georgetown.edu/cgi-bin/pro/entry_pro?id=PR:000027213) | Toll/interleukin-1 receptor domain-containing adapter protein phosphorylated form | [P58753](http://www.uniprot.org/uniprot/P58753) | [PR:000027214](http://pir.georgetown.edu/cgi-bin/pro/entry_pro?id=PR:000027214) | none | None |

For each of the three nonprotein molecules involved in forming TLR3 and TLR4 complexes, its name and identifier in the ChEBI reference database is given. For each of the proteins involved in these complexes, PRO name and the UniProt and PRO identifiers for its mouse and human forms are given. In this version of the table published in the paper, hyperlinks have been embedded in all database identifiers, allowing direct access to these resources. The two tables are otherwise identical.
