# Supplementary material for: Toll-Like Receptor Signaling in Vertebrates: Testing the Integration of Protein, Complex, and Pathway Data in the Protein Ontology Framework
Source: PLoS One. 2015 Apr 20;10(4):e0122978. doi: 10.1371/journal.pone.0122978 (PMC4404318; doi:10.1371/journal.pone.0122978)
Supplement: S4 Table — (DOCX) [file pone.0122978.s004.docx]

**S4 Table. Annotation of species-specific functions of MD2:TLR4 complexes**

| **MD2:TLR4 complex** | **Annotation** | Evidence Annotation **An Ev Evidence** |
| --- | --- | --- |
| Mouse PR:000036005 | participates_in Toll-like receptor 4 signaling pathway (GO:0034142) | Kim et al. 2007 |
| Human PR:000036004 | participates_in Toll-like receptor 4 signaling pathway (GO:0034142) | [REACT_6894](http://www.reactome.org/cgi-bin/control_panel_st_id?ST_ID=REACT_6894) |
| Chicken PR:000037473 | NOT participates_in MyD88-independent toll-like receptor signaling pathway (GO:0002756) | Keestra & van Putten 2008, Haddadi et al. 2013 [REACT_25089](http://www.reactome.org/cgi-bin/control_panel_st_id?ST_ID=REACT_25089.1) |

In this version of the table published in the paper, hyperlinks have been embedded in all database identifiers, allowing direct access to these resources. The two tables are otherwise identical.
